# MALE BREAST CANCER IN OLDER ADULTS BELONGING TO THE LOWER-INCOME GROUP

**DOI:** 10.1093/geroni/igad104.2471

**Published:** 2023-12-21

**Authors:** Esha Chakravarty

**Affiliations:** Calcutta Metropolitan Institute of Gerontology, Mitcham, England, United Kingdom

## Abstract

Male breast cancer is a rare type of cancer constituting around 1% of male malignancies. Cases of 21 elderly (aged>60) patients diagnosed with breast cancer were studied with respect to age of onset, treatment choices, access to care, ability to perform activities of daily living (ADL) from CMIG, resource centre on ageing in east India. All 21 patients belonged to lower income group in society and the study was conducted over a 5 year period of 2018-2022. Primary symptom leading to diagnosis was self-detection of lump and the median time of diagnosis from onset of symptoms was 6 months. Twenty of the 21 patients were diagnosed with Stage III or Stage IV breast cancer and 1 patient was diagnosed with Stage I cancer. The average age of diagnosis of the patients was found to be 69 years. The main method of treatment was surgery for patient diagnosed in Stage I and surgery followed by chemotherapy and radiation therapy for the rest. None of the patients underwent hormone therapy and information regarding presence of hormone receptors was not available in all cases. Five of the 21 cases reported metastasis- 4 in bone and 1 in lung. Spouse or adult children were found to be caregivers with no access to trained caregivers or counsellors. All patients showed moderate decline in motor function and ADL after surgery alongwith susceptibility to fall or injury. Free hand exercises showed slight improvement. Further studies can attract state funding and influence heath policies for lower income groups.

